# Effects of Simulated Nitrogen Deposition on the Physiological and Growth Characteristics of Seedlings of Two Typical Subtropical Tree Species

**DOI:** 10.3390/plants14142153

**Published:** 2025-07-11

**Authors:** Zhenya Yang, Benzhi Zhou

**Affiliations:** 1 Zhejiang Academy of Forestry, Hangzhou 310023, China; yangzhenya1234@163.com; 2 Northwest Zhejiang Bamboo Forest Ecosystem Positioning Observation and Research Station, National Forestry and Grassland Administration, Hangzhou 310023, China; 3 Research Institute of Subtropical Forestry, Chinese Academy of Forestry, Hangzhou 311400, China

**Keywords:** *Phyllostachys edulis*, *Cunninghamia lanceolata*, nitrogen deposition, root architecture, physiological growth

## Abstract

Amid global environmental change, the intensification of nitrogen (N) deposition exerts critical impacts on the growth of forest vegetation and the structure and function of ecosystems in subtropical China. However, the physiological and growth response mechanisms of subtropical tree species remain poorly understood. This study explored adaptive mechanisms of typical subtropical tree species to N deposition, analyzing biomass accumulation, root plasticity, and nutrient/photosynthate allocation strategies. One-year-old potted seedlings of *Phyllostachys edulis* (moso bamboo) and *Cunninghamia lanceolata* (Chinese fir) were subjected to four N-addition treatments (N0: 0, N1: 6 g·m^−2^·a^−1^, N2: 12 g·m^−2^·a^−1^, N3: 18 g·m^−2^·a^−1^) for one year. In July and December, measurements were conducted on seedling organ biomass, root morphological and architectural traits, as well as nutrient elements (N and phosphorus(P)) and non-structural carbohydrate (soluble sugars and starch) contents in roots, stems, and leaves. Our results demonstrate that the Chinese fir exhibits stronger tolerance to N deposition and greater root morphological plasticity than moso bamboo. It adapts to N deposition by developing root systems with a higher finer root (diameter ≤ 0.2 mm) ratio, lower construction cost, greater branching intensity and angle, and architecture approaching dichotomous branching. Although N deposition promotes short-term biomass and N accumulation in both species, it reduces P and soluble sugars contents, leading to N/P imbalance and adverse effects on long-term growth. Under conditions of P and photosynthate scarcity, the Chinese fir preferentially allocates soluble sugars to leaves, while moso bamboo prioritizes P and soluble sugars to roots. In the first half of the growing season, moso bamboo allocates more biomass and N to aboveground parts, whereas in the second half, it allocates more biomass and P to roots to adapt to N deposition. This study reveals that Chinese fir enhances its tolerance to N deposition through the plasticity of root morphology and architecture, while moso bamboo exhibits dynamic resource allocation strategies. The research identifies highly adaptive root morphological and architectural patterns, demonstrating that optimizing the allocation of elements and photosynthates and avoiding elemental balance risks represent critical survival mechanisms for subtropical tree species under intensified N deposition.

## 1. Introduction

Nitrogen (N) deposition, a critical manifestation of global change, profoundly influences the growth of forest vegetation and the structure and function of ecosystems [1]. Human activities such as fossil fuel combustion and chemical fertilizer application have increased atmospheric N deposition by more than tenfold over the past century [2]. Among them, the subtropical region of China is a hotspot for N deposition, with an annual average N deposition exceeding 65 kg N ha^−1^yr^−1^ [3]. Scientists predict that the subtropical region will become one of the areas with the most severe atmospheric N deposition globally in the coming decades, and tremendous changes will occur in the terrestrial ecosystems and related biogeochemical cycling processes in this region [4]. N is an indispensable nutrient element for plant growth and productivity. Changes in soil N availability caused by N deposition will directly affect plant functional traits, metabolic processes, and production performance, serving as a crucial driving force for changes in ecosystem structure and function [5]. Elucidating plant physiological and growth response strategies to N deposition is crucial for informing soil nutrient management strategies and maintaining the stability of forest ecosystems under ongoing N deposition.

Plants exhibit varying demands and tolerance levels for N, leading to dual effects of N deposition on plant growth. N is often the primary limiting factor for plant growth, even in low-phosphorus (P) regions such as subtropical areas in China [6]. N deposition can alleviate soil nutrient limitations in low-N regions and enhance net photosynthetic rates by promoting leaf chlorophyll biosynthesis and improving the activity of photosynthesis-associated enzymes, thus facilitating plant growth [7]. However, the promoting effect of N deposition exhibits a “saturation effect” when N input exceeds the threshold of plant demand, disrupting the balance of N metabolism in plants and thereby inhibiting plant growth [8]. Additionally, excessive N deposition can cause issues such as soil acidification, imbalance of soil nutrient elements, and decline in microbial biodiversity, indirectly hindering plant growth [9,10,11]. Although low-level N deposition can promote plant growth in the short term, it may lead to excessive consumption of macronutrients other than N (e.g., carbon and P), micronutrients, and photosynthetic products. Elements such as carbon and phosphorus may become limiting factors for plant growth under N deposition. Therefore, plants with reasonable and efficient resource allocation capabilities can generally benefit in the long term under N deposition. The resource optimization hypothesis posits that under conditions of exogenous N input, plants tend to allocate more carbon and N to the organs that limit their growth, thereby promoting their own growth and enhancing adaptability [12]. For example, studies have demonstrated that N deposition can promote certain plants to preferentially allocate photosynthetic products to leaves and stems rather than roots, thereby favoring aboveground photosynthetic carbon fixation over root nutrient absorption [13]. However, numerous conflicting results also exist. For instance, geophytes with specialized underground storage organs and some invasive plants with strong root competitive abilities tend to invest resources preferentially in roots [14,15].

Plants rely on their morphological and physiological plasticity to adapt to environmental changes caused by N deposition. For example, under low-level N deposition, plants enhance root nutrient absorption capacity by increasing the number of lateral roots and root surface area, or forming a “shallow-rooted” architecture by increasing root density in surface soil to promote N foraging in high-N soil layers [16,17]. Plants can also sense high-N signals, regulate local auxin content in roots, inhibit lateral root branching and growth, and promote taproot elongation to form a “deep-rooted” architecture, thereby adapting to the inhibitory effects of long-term high-N input [18,19,20]. Beyond analyzing root quantity and length, introducing fractal and topological concepts to analyze and quantify root architectural patterns via branching modes and intensities can provide deeper insights into the adaptive mechanisms of plants to N deposition [21,22].

The ability of plants to convert soil nitrogen into biomass (nitrogen use efficiency, NUE) does not simply increase with rising soil N content. N deposition alters NUE through dual mechanisms: short-term moderate N enrichment can enhance NUE by promoting fine-root proliferation, while long-term excessive N input leads to decreased NUE due to soil elemental imbalance (e.g., low soil C/N ratio) [23]. Additionally, plant NUE responses to N input are modulated by multiple variables, including soil N form, plant developmental stage, root architecture, and soil nutrient status [24,25]. For instance, moso bamboo exhibits significantly higher NUE for ammonium nitrogen than nitrate nitrogen [26]. Compared with C4 plants, C3 plants (e.g., wheat and rice) exhibit significantly higher growth sensitivity to N deposition [27]. Woody plants, with their more complex root systems and longer life cycles, exhibit slower and more cumulative responses to N deposition compared to herbaceous plants [28,29]. Differences in response strategies of coexisting tree species to N deposition alter interspecific competition patterns, driving forest succession toward communities dominated by high-N-adapted species, which in turn results in reduced species diversity and simplified community structures. In-depth analysis of physiological and growth responses of coexisting plants to N deposition is of great significance for maintaining forest ecological security under global change. Moso bamboo (*Phyllostachys edulis* (Carr.) H. de Lehaie f. edulis) and Chinese fir (*Cunninghamia lanceolata*) are both important forest resources in subtropical China, possessing significant economic and ecological values. Their mixed forests are also typical stand types in subtropical China [30]. Due to their distinct differences in growth rates, reproductive modes, and root morphological structures, their adaptive strategies to N deposition may vary significantly. However, studies on the adaptive mechanisms of Chinese fir and moso bamboo to N deposition remain limited. This research aims to elucidate the adaptive strategies of these two co-occurring species to N deposition by examining their root morphological architecture, biomass allocation, photosynthate partitioning, and nutrient element distribution patterns at different growth stages under varying N deposition intensities.

## 2. Materials and Methods

### 2.1. Experimental Setup

This experiment was conducted at the Research Institute of Subtropical Forestry, Chinese Academy of Forestry (119°57′ E, 29°48′ N), located in Zhejiang Province. The study site exhibited a typical subtropical monsoon climate, featuring a 307-day frost-free period, an average annual sunshine duration of 1663.2 h, a mean relative humidity of 70.3%, and an altitude of 93 m.

The experimental seeds of each species were sourced from the same parent plant. The parent plants of moso bamboo and Chinese fir were located at the Da Jing Town Forestry Center (Guilin, Guangxi Zhuang Autonomous Region) and Changle Forestry Center (Hangzhou, Zhejiang Province), respectively. Seeds of the two species, soaked in deionized water, were cultivated in a temperature incubator until germination. Seedlings with uniform bud length were transplanted into seedling trays, which are plug trays with 32 cells, with one seedling planted in each cell. Each cell has a depth of 11 cm and a square upper aperture with a side length of 6 cm. The substrate for filling the trays consisted of peat, vermiculite, and perlite at a 2:1:1 ratio. In April, seedlings of each species with consistent height and basal diameter were transplanted into plastic pots with an internal diameter of 25 cm and a height of 27 cm. Preliminary tests confirmed that the containers were sufficiently large to allow unimpeded growth of the two tree seedlings for one year. Each pot was filled with 6 kg of soil. The soil (pH 4.91) had the following properties: organic carbon (18.7 g·kg^−1^), total N (0.86 g·kg^−1^), total P (0.26 g·kg^−1^), total potassium (11.2 g·kg^−1^), hydrolyzable N (85.13 mg·kg^−1^), available P (4.15 mg·kg^−1^), and available potassium (65.73 mg·kg^−1^). The soil was collected from mixed forests of moso bamboo and Chinese fir in subtropical regions of China to replicate the natural growth conditions of the two species. Soil moisture was precisely regulated using a combination of soil weighing and a high-precision soil moisture monitoring system (IMKO GmbH, Trime-pico AZS-100, Ettlingen, Germany), and consistently maintained at 80–85% of the maximum field water-holding capacity.

**Figure 1:**
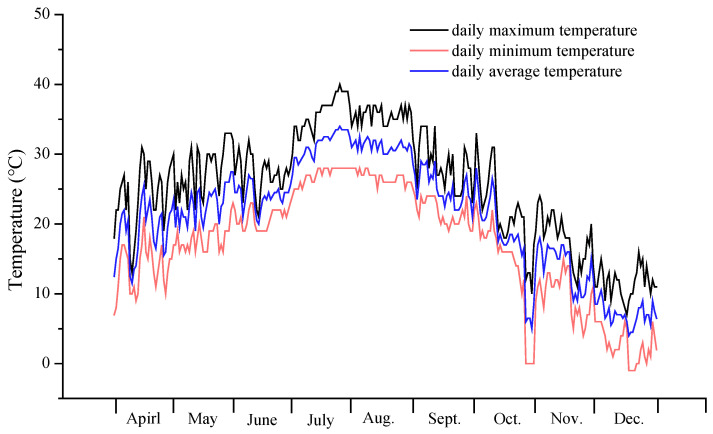
Temperature at the test site during the test period.

Forty seedlings each of moso bamboo and Chinese fir were allocated into four groups, with 10 seedlings per group, each group subjected to one of the four distinct N deposition treatments. NH_4_NO_3_ was dissolved in water and sprayed onto the seedlings to simulate N deposition. The N addition amounts for the four treatments were 0 (N0), 300 mg·pot^−1^ (N1), 600 mg·pot^−1^ (N2), and 1200 mg·pot^−1^ (N3). The N addition amounts for the four treatments, converted to N deposition equivalents, were 0, 6 g·m^−2^·a^−1^, 12 g·m^−2^·a^−1^, and 24 g·m^−2^·a^−1^. The N addition rates were determined based on the regional N deposition levels and the application rates reported in comparable studies [31]. The annual requirement of NH_4_NO_3_ was divided into three equal portions and applied three times at 15-day intervals. Each portion of NH_4_NO_3_ was dissolved in 200 mL of deionized water before being uniformly sprayed onto the soil in the pots. Consistent lighting and temperature regimes were maintained in the greenhouse throughout the experiment. The temperature change at the test site during the test period is shown in Figure 1.

### 2.2. Harvest and Measurements

**Figure 2:**
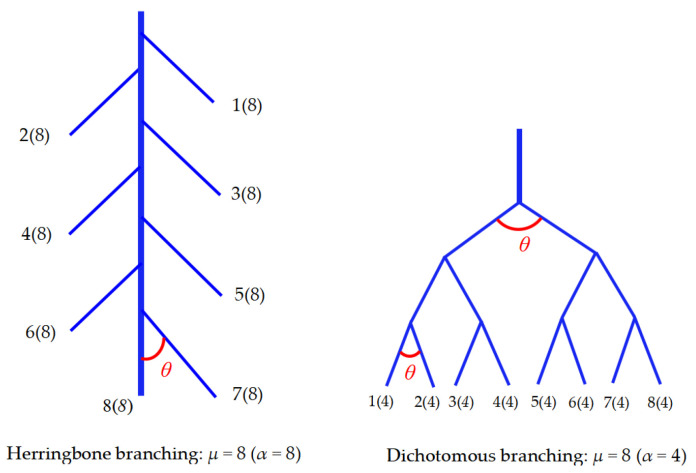
Diagram of root topology classification.

In July and December of the same year, sampling was performed on 10 seedlings in each treatment group. Five seedlings were sampled each time as five replicates. The leaves, stems, and roots were separated using scissors. The soil was then carefully shaken off to avoid damaging the root tissues and architecture, followed by collecting all residual roots through a 2 mm sieve. The roots, stems, and leaves were sealed in self-sealing bags, stored in an ice box at 0–2 °C, and transported to the laboratory. The roots were meticulously rinsed with water and gently blotted dry with absorbent paper, then scanned at a high resolution of 500 dpi using a double-sided scanner (Regent Instruments Inc., WinRhizo Pro, Québec, QC, Canada). The root images were subsequently analyzed using WinRhizo software (version 2.0) to precisely quantify root morphological parameters. These included root length (RL, cm), root surface area (RSA, cm^2^), root average diameter (RD, mm), root tip number (RT, pieces·plant^−1^), and number of root links. Additionally, root architectural traits such as fractal dimension (FD) and root branching angle (RBA, °) were determined. The FD was computed via the box-counting method implemented in the WinRhizo software [32]. Root lengths of eleven diameter grades (including 0–0.2 mm, 0.2–0.4 mm, 0.4–0.6 mm, … >2 mm) were also derived using WinRhizo software. The root length ratio (RLR) was calculated by dividing the root length in each diameter grade (rl) by the total root length. In this study, we also introduced the concept of topology and calculated the root topological index (T1) using the following formula. Figure 2 illustrates two typical root topologies, herringbone branching and dichotomous branching, with TI values of 1 and 0.5, respectively.

TI = lg (α)/lg (µ)

Root tips were designated as external nodes, while branch points were termed internal nodes. Correspondingly, root segments between any two nodes were defined as links. Links not terminating at a terminal point within the root architecture were classified as internal links, with the remainder designated as external links. External links were further categorized as external–external (EE) when extending from other external links or external–internal (EI) when originating from internal links. The altitude (α) was defined as the number of internal links along the longest pathway from the root collar to an external root tip. Concurrently, the magnitude (µ) represented the total number of external links in the root system, equivalent to the RT [33].

Roots, stems, and leaves were subjected to a 30-min inactivation treatment at 105 °C. Samples were then dried at a constant temperature of 65 °C until constant weight was reached to determine the dry weight (biomass) of each tissue. Specific root length (SRL) was calculated as the ratio of total root length to root dry weight. N and P contents in roots, stems, and leaves were measured using the H_2_O_2_-H_2_SO_4_ digestion method and vanadium molybdenum yellow colorimetry, respectively [34]. Contents of non-structural carbohydrates (NSCs), including soluble sugars (SS) and starch (ST), were determined using Anthrone colorimetry [35]. The ratio of nutrient elements accumulation is the ratio of the nutrient accumulation in a single organ to that in the entire plant.

### 2.3. Statistical Analysis

The significance of differences between parameters was analyzed using SPSS software (Version 20.0; IBM SPSS Statistics, Armonk, NY, USA). A three-way analysis of variance (ANOVA) was performed to assess the main effects and interaction effects of N-addition treatments, periods, and species on the indicators. Prior to ANOVA, data normality and homogeneity of variances were verified using the Shapiro–Wilk test and Levene’s test, respectively. For the effect of N-addition treatments, one-way ANOVAs were conducted, followed by Tukey’s honest significant difference (HSD) post-hoc tests to identify significant differences among treatment levels.

## 3. Results

### 3.1. Effects of N Deposition on the Biomass Allocation of Moso Bamboo and Chinese Fir Seedlings

Treatments, periods, and species all significantly affected stem biomass, leaf biomass, and root–shoot ratio (Table 1). Moreover, significant interaction effects were observed in all pairwise combinations (Treatments × periods, Treatments × species, periods × species) and the three-way interaction (Treatments × periods × species). Root biomass, treatments, periods, and species also exerted significant main effects, yet a significant interaction effect was only detected between periods and species.

**Figure 3:**
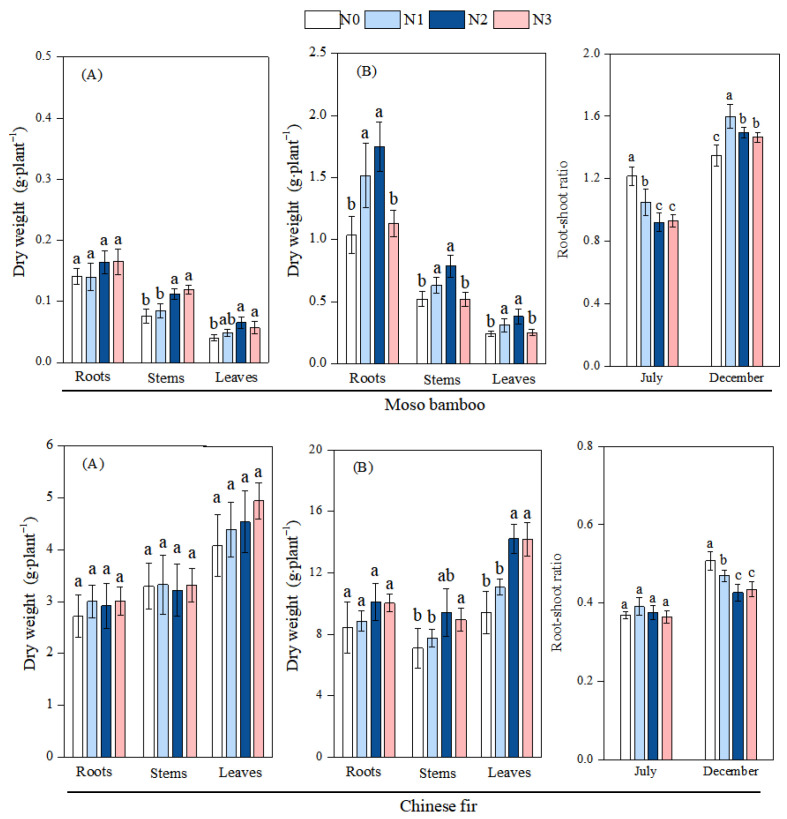
Effects of N deposition on the biomass allocation of moso bamboo and Chinese fir seedlings. Note: a, b, c—different letters indicate significant differences among different treatments (*p* < 0.05). The values in the bar chart are the means and standard errors. (**A**): July, (**B**): December.

For the first measurement in July, with increasing N addition, the leaf and stem biomass of moso bamboo increased significantly, while its root–shoot ratio decreased significantly. In Chinese fir, no significant differences were observed in biomass across various organs or in root–shoot ratios among treatments. For the second measurement in December, with increasing N addition, the biomass of roots, stems, and leaves, as well as the root–shoot ratio, of moso bamboo exhibited a trend of first increasing and then decreasing. The biomass of roots, stems, and leaves under the N1 and N2 treatments was significantly higher than that under the N0 and N3 treatments. Additionally, the root–shoot ratio of moso bamboo under N-addition treatments was significantly higher than that under the N0 treatment. For Chinese fir, N addition had no significant effect on root biomass but significantly enhanced leaf and stem biomass, leading to a notable decrease in root–shoot ratios (Figure 3).

### 3.2. Effects of N Deposition on the Root Morphology and Architecture of Moso Bamboo and Chinese Fir Seedlings

Treatments, periods, and species all significantly affected RL, RSA, SRL, and RT (Table 1). Significant interaction effects on RSA and RT were detected in all pairwise factor combinations (treatments × periods, treatments × species, periods × species), as well as in the three-factor interaction (treatments × periods × species). Periods and species significantly affected RD and FD; treatments and species significantly affected TI. Furthermore, the periods × species interaction significantly affected RD, RL, and FD, while the treatments × species interaction significantly affected RD.

**Figure 4:**
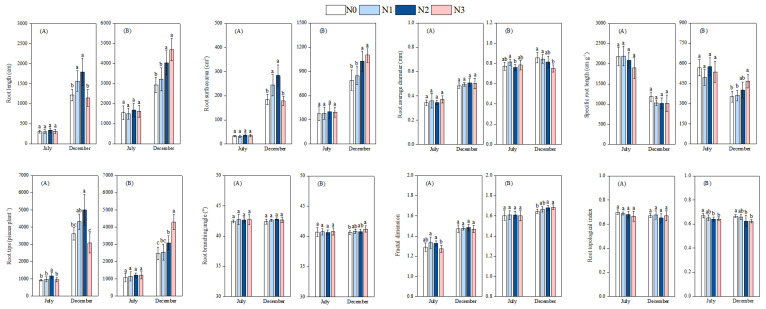
Effects of N deposition on the root morphology and architecture of moso bamboo and Chinese fir seedlings. Note: a, b, c—different letters indicate significant differences among different treatments (*p* < 0.05). The values in the bar chart are the means and standard errors. (**A**): moso bamboo, (**B**): Chinese fir.

Regarding root morphology, for the first measurement in July, N addition had no significant effects on RL, RSA, RD, and SRL in moso bamboo and Chinese fir. For the second period in December, with increasing N addition, RL and RSA of moso bamboo exhibited a trend of first increasing and then decreasing, which both reached their peak values under the N2 treatment. By contrast, no significant differences in RD and SRL of moso bamboo were observed among different treatments. For the second period in December, with increasing N addition, RL, RSA, and SRL of Chinese fir showed a gradual upward trend, while RD presented a gradual downward trend. The RL and RSA of Chinese fir under the N2 and N3 treatments were significantly greater than those under the N0 and N1 treatments (Figure 4).

Regarding root architecture, for the first measurement in July, with increasing N addition, the RT and FD of moso bamboo exhibited a trend of first increasing and then decreasing, while RBA and TI showed no significant changes. For Chinese fir, the TI of roots gradually decreased with the increase in N addition, whereas FD, RT, and RBA showed no significant changes. For the second period in December, the RT of moso bamboo showed a trend of first increasing and then decreasing with the increase in N addition. The RT and FD of Chinese fir gradually increased. The RBA of Chinese fir under the N3 treatment was significantly greater than that under N0 treatment, and the TI under N2 and N3 treatments was significantly lower than that under N0 treatment (Figure 4).

**Figure 5:**
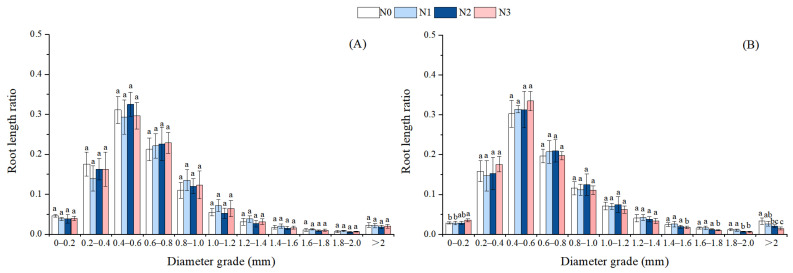
Effects of N deposition on the root length ratio of Chinese fir seedlings. Note: a, b, c—different letters indicate significant differences among different treatments (*p* < 0.05). The values in the bar chart are the means and standard errors. (**A**): July, (**B**): December.

Due to the significant effects of different treatments on the RD of Chinese fir, the RLR across different diameter grades was analyzed. For the first measurement in July, N-addition treatments had no significant effects on RLR across different diameter grades of Chinese fir. For the second period in December, with increasing N addition, the RLR of the 0–0.2 mm diameter grade of Chinese fir gradually increased, while the RLRs of the 1.4–1.6 mm, 1.6–1.8 mm, 1.8–2 mm, and >2 mm diameter grades gradually decreased. Compared with the N0 treatment, the N3 treatment significantly increased the RLR of the 0–0.2 mm diameter grade and significantly decreased the RLRs of the 1.4–1.6 mm, 1.6–1.8 mm, 1.8–2 mm, and >2 mm diameter grades (Figure 5). Throughout the entire growing season, there were no significant differences in RLR of moso bamboo among treatments, so the results are not presented.

### 3.3. Effects of N Deposition on the Allocation of Nutrient Elements and Non-Structural Carbohydrates of Moso Bamboo and Chinese Fir Seedlings

Treatments, periods, and species all significantly affected leaves N, stems N, roots N, leaves P, stems P, roots P, stems N/P, and roots N/P. All pairwise interactions among the three factors significantly affected leaves N, stems N, leaves N/P, and roots N/P. The interaction between treatments and periods significantly affected roots N and stems N/P (Table 1).

For the first measurement in July, N addition increased the N content in roots, stems, and leaves of Chinese fir, while decreasing P content in its roots, stems, leaves, as well as in stems and leaves of moso bamboo. For moso bamboo, no significant differences were observed in root P contents or in N contents of roots, stems, and leaves across treatments. There was no significant difference in P content in the leaves of Chinese fir between the N0 and N1 treatments. N addition enhanced the N/P ratio in roots, stems, and leaves of both moso bamboo and Chinese fir. For the second period in December, N addition significantly decreased P content in leaves and stems of moso bamboo, as well as in roots, stems, and leaves of Chinese fir. Conversely, N addition significantly increased N content and N/P ratios in roots, stems, and leaves of both species. N addition had no significant effect on P content in the roots of moso bamboo throughout the study period (Table 2).

Treatments, periods, and species all significantly affected leaves SS, leaves ST, stems ST, roots ST, leaves SS/ST, stems SS/ST, and roots SS/ST. Additionally, treatments and species significantly affected stems SS and roots SS. All pairwise interactions among the three factors significantly affected stems SS, leaves SS/ST, and roots SS/ST. Additionally, the treatments × periods and treatments × species interactions significantly affected roots SS and stems SS/ST (Table 1).

For the first measurement in July, compared with the N0 treatment, N-addition treatments (N1, N2, and N3) significantly reduced the content of SS in leaves and stems of moso bamboo, while increasing ST content in its roots, stems, and leaves. No significant effect was observed on the SS content in moso bamboo roots. N-addition treatments (N2 and N3) significantly reduced the content of SS in roots and stems of Chinese fir, while increasing ST content in its roots and leaves. No significant effect was observed on the SS content in Chinese fir leaves. The results of December were similar to those of July. In both periods, with increasing N addition, the SS/ST ratio in roots, stems, and leaves of moso bamboo and Chinese fir showed a gradual decreasing trend. Compared with the first period, the effects of the N1 treatment on ST and SS contents in the roots of Chinese fir were more significant in the second period (Table 3).

### 3.4. Effects of N Deposition on the Nutrient Accumulation Ratio in Different Organs of Moso Bamboo and Chinese Fir Seedlings

Treatments, periods, and species all significantly affected the N accumulation ratios in leaves and roots, as well as the P accumulation ratios in leaves, stems, and roots. Significant interaction effects on P accumulation ratios in leaves, stems, and roots were detected in all pairwise factor combinations (treatments × periods, treatments × species, periods × species), as well as in the three-factor interaction (treatments × periods × species). The interaction between treatments and periods significantly affected the N accumulation ratios in stems and roots (Table 1).

**Figure 6:**
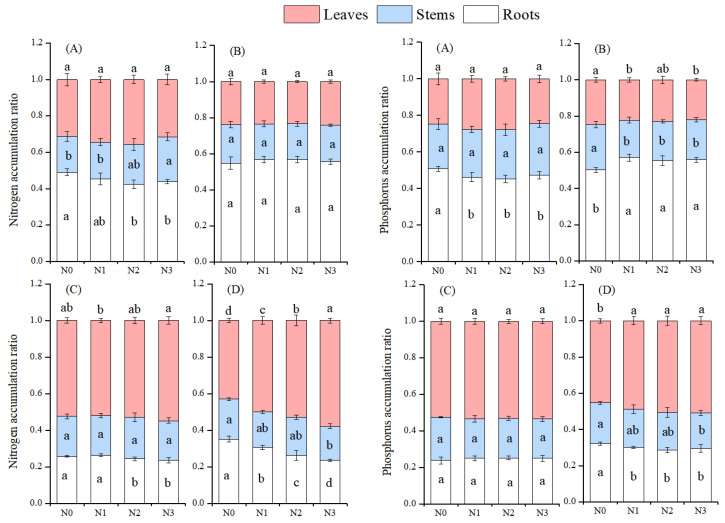
Effects of N deposition on the nutrient accumulation ratio in different organs of moso bamboo and Chinese fir seedlings. Note: a, b, c—different letters indicate significant differences among different treatments (*p* < 0.05). The values in the bar chart are the means and standard errors. (**A**): data of moso bamboo in July, (**B**): data of moso bamboo in December, (**C**): data of Chinese fir in July, (**D**): data of Chinese fir in December.

For the first measurement in July, with increasing the N addition, the accumulation ratios of N and P in moso bamboo roots decreased, while the N accumulation ratio in stems increased. In Chinese fir, the N accumulation ratio in roots decreased, whereas that in leaves increased. For the second measurement in December, with increasing the N addition, the P accumulation ratio in moso bamboo roots increased, while those in stems and leaves decreased. In Chinese fir, the accumulation ratios of N and P in leaves increased, while those in stems and roots decreased (Figure 6).

## 4. Discussion

### 4.1. Responses of Biomass Allocation to N Deposition

Plants exhibit regulatory capabilities in biomass allocation strategies, enabling them to more efficiently acquire resources under diverse soil nutrient conditions and thereby maintain growth, reproduction, and survival [36]. In this study, N addition promoted biomass accumulation in both plant species, consistent with previous findings [7]. High-level N input inhibited the growth of moso bamboo but not Chinese fir, suggesting that moso bamboo is more sensitive to high N or has a lower N demand than Chinese fir. These results align with previous reports showing that high N deposition can inhibit plant growth [8]. Due to the competitive mechanism between ions, excessive N input hinders plants’ absorption of other elements (e.g., P and potassium), leading to imbalanced elemental ratios, inhibition of N metabolic processes and photosynthesis, and, consequently, suppression of plant growth [37]. For Chinese fir, N addition significantly decreased the root–shoot ratio and increased shoot biomass in the second half of the growing season. This indicates that in N-rich soils, Chinese fir prioritizes aboveground growth to enhance whole-plant carbon fixation capacity, rather than allocating resources to roots for consumption or storage. For moso bamboo, N addition decreased the root–shoot ratio in the first half of the growing season but increased it in the second half. Obviously, moso bamboo employs different strategies to balance the growth of aboveground parts and roots at distinct stages to adapt to N deposition. Specifically, moso bamboo prioritizes aboveground growth in the first half of the growing season and shifts to root growth in the second half. This finding aligns with the “Relative Growth Relationship Hypothesis” proposed in previous studies, which posits that plants adjust the relative growth rates of different organs in response to soil nutrient variations to achieve optimal resource allocation [38]. Some studies have also found that N-addition treatments exhibit opposite effects on the root-shoot ratio at different stages of plant growth and development, which may be attributed to the different growth priorities of plants during distinct growth phases [39,40]. This result can guide the fertilization method of moso bamboo, such as applying foliar N fertilizer before July and applying N fertilizer around the root between July and December to achieve the goal of precise nutrient supply. For the other two subtropical tree species that often coexist with moso bamboo and Chinese fir, their tolerance to nitrogen deposition is even lower. Song et al. found that when the N deposition dose reached 12 g·m^−2^·a^−1^, the root length and root biomass of two Pinus massoniana clones significantly decreased [41]. The response of biomass allocation and leaf nutrient elements of Schima superba to N deposition is similar to that of Chinese fir, but when the N deposition increases to 15 g·m^−2^·a^−1^, its biomass accumulation is significantly inhibited [42].

### 4.2. Responses of Root Morphology and Architecture to N Deposition

In this study, root elongation of both plant species did not respond to N addition until the second half of the growing season, which aligns with previous findings that plant roots exhibit delayed responses to N input compared to aboveground parts [14]. High N (N3) input significantly inhibited root growth in moso bamboo, a conclusion also reported in prior studies. This may be because excessive N input can induce ammonium stress in plants, leading to callose accumulation in roots, which in turn inhibits the transport and unloading of sucrose in root phloem, ultimately resulting in restricted root growth [43]. N addition increased the root length of Chinese fir by 60.2%, which was significantly higher than the 47.2% increase in moso bamboo. The root growth of Chinese fir was not inhibited by high N, as observed in moso bamboo. Instead, it produced more fine roots, reducing root diameter and root construction costs to adapt to high N conditions. This suggests that the roots of Chinese fir exhibit stronger adaptability to high N deposition compared to moso bamboo, characterized by a higher degree of morphological plasticity in response to N input. Specifically, Chinese fir produced more fine roots with lower costs and increased the ratio of absorptive-function roots to improve the return on carbon investment in the root system. Xu et al. found similar conclusions that N deposition at 30 g·m^−2^·a^−1^ significantly increased the finer root biomass of the 0–0.5 mm diameter of *Populus deltoides* [44]. Previous studies have also drawn similar conclusions, suggesting that the roots become thinner under high-N conditions because more carbon is used for the growth of the above-ground parts [45]. In this study, the decrease in SS content in the roots and the root-shoot ratio of Chinese fir under high-N conditions also confirms this view. Some studies suggest that root thinning under high N may be attributed to changes in the abundance of proteins involved in cell expansion and lignin biosynthesis [46].

In terms of root architecture, N addition can promote root branching in Chinese fir and moso bamboo to varying degrees. Similar studies have found that ammonium N supply can stimulate the accumulation of shoot-derived auxin in root vasculature and promote lateral root emergence to build a highly branched root system [47]. In the first half of the growing season, N addition increased the branching intensity of moso bamboo roots to a certain extent, but this promoting effect disappeared in the second half of the growing season. This indicates that N addition only accelerated root branching rather than altering the final branching intensity. However, Chinese fir showed an opposite trend. Combined with the results of the root–shoot ratio, the reason for this phenomenon may be the different growth peak periods of the root systems of the two species. The increase in RBA of Chinese fir indicates that N addition drives roots to forage for nutrients in the topsoil. Previous studies have also found that high N can cause plants to form shallower root systems [17]. Chinese fir adapts to elevated soil N by modifying its branching pattern to reduce T1, forming a root architecture approaching dichotomous branching. This architectural strategy is better suited for fast-growing species to rapidly expand and forage for nutrients under nutrient-rich conditions [32]. Additionally, insufficient carbon supply to roots may play a role, as dichotomous root systems entail lower construction costs and enable quicker soil space occupation [33].

### 4.3. Responses of Nutrient Element and Non-Structural Carbohydrate Allocation to Different Soil N Deposition

In this study, N addition increased the N content in various organs of Chinese fir and moso bamboo but significantly decreased the P content. This has led to a significant increase in the N/P ratio in various organs. For example, the leaf N/P ratio of Chinese fir increased from 12.01 to 35.41. This indicates that N deposition can shift the growth pattern of Chinese fir and moso bamboo from N-limited (with an N/P ratio less than 14) to P-limited (with an N/P ratio greater than 16) [34]. Conversely, some previous studies have found that high N input can enhance rhizosphere soil P availability by inducing root secretion of organic acids and phosphatases and activating the expression of P transporter genes to improve P absorption and transport capacity [48,49]. However, the synergistic absorption effect of N and P was not observed in this study. This may be because the ion competition mechanism of the two plants dominated during nutrient absorption [34]. For example, it has been confirmed that competition occurs between phosphate ions (H_2_PO_4_^−^/HPO_4_^2−^) and nitrate ions (NO_3_^−^) in anion channels [50]. In addition, the increase in the growth of various organs after N addition will also lead to a large consumption of P. This indicates that although N deposition promotes the growth of the two plants in the short term, it causes an imbalance of the N/P ratio, which is adverse to the long-term growth and development of plants. Notably, P content in the stems and leaves of moso bamboo significantly decreased under N-addition treatments, while no significant decline was observed in roots. This indicates that under P-deficient conditions caused by N deposition, moso bamboo preferentially allocates P to roots to ensure P absorption. Previous studies have found that under P-deficient conditions, *Arabidopsis thaliana* reduces the stability of P transporters, hinders P transport to aboveground parts, and thus retains more P in roots [51].

From the results of nutrient allocation ratios, under the influence of N deposition, moso bamboo tended to allocate more N to stem growth in the first half of the growing season, while Chinese fir preferred to allocate more N to leaves throughout the growing season. Wang’s study drew similar conclusions, finding that N deposition significantly increased the aboveground N pool of plants, but had little effect on roots [52]. In the second half of the growing season, when moso bamboo and Chinese fir faced P deficiency, they exhibited different strategies: moso bamboo focused on allocating P to roots, whereas Chinese fir prioritized leaves. In the first half of the growing season, N addition caused a decrease in the P accumulation ratio in moso bamboo roots, possibly due to the preferential aboveground growth during this period. Compared with moso bamboo, Chinese fir exhibits faster growth, higher N demand, and greater NUE. This enables it to show more positive root morphological and architectural responses, as well as larger biomass increment, under high-dose N deposition. For moso bamboo, the N addition at P3 level exceeds its tolerance limit, inhibiting root N absorption and conversion capacity, reducing NUE, and thus leading to negative responses in biomass accumulation and root growth. Su et al. reached similar conclusions, finding that when the nitrogen application rate in maize exceeded 225 kg·ha^−1^, both biomass and NUE shifted from increasing to decreasing [53].

The findings of this study provide two key implications for forest ecological management under N deposition. First, in moso bamboo forests subjected to high N deposition (N deposition > 12 g·m^−2^·a^−1^), applying P fertilizer or biochar is recommended to maintain nutrient balance, enhance NUE, and mitigate the risk of long-term growth inhibition caused by nutrient imbalance. Second, in terms of species configuration, for moso bamboo forests under high N deposition, it is recommended to interplant tree species with high N consumption and NUE, such as Chinese fir, or species preferentially absorbing nitrate nitrogen, such as blue oak (*Cyclobalanopsis glauca*), so as to alleviate the inhibitory effect of high N on the growth of moso bamboo.

### 4.4. Responses of Non-Structural Carbohydrate Allocation to Different Soil N Deposition

NSC are crucial photosynthetic products that support plant growth, metabolism, and a cascade of physiological processes [54]. In this study, N addition promoted a decrease in SS content and an increase in ST content in moso bamboo and Chinese fir, leading to a reduction in the SS/ST. This indicates that N deposition promotes the conversion of SS to ST. In studies on sweet potatoes, N addition has been found to promote the expression of genes corresponding to glucose pyrophosphorylase and starch synthase, thereby facilitating the conversion of SS to ST [55]. The rapid plant growth exacerbates the decrease in SS due to increased consumption. Notably, SS in Chinese fir leaves and moso bamboo roots did not decrease, indicating that the two species exhibit distinct allocation strategies under SS limitation: moso bamboo prioritizes allocating SS to roots, whereas Chinese fir allocates it to leaves. In wheat and crabapple, it has also been found that N levels can alter the allocation priority of SS among different plant organs by influencing the activities of sugar transporters and metabolic enzymes, as well as the expression of their corresponding genes [56,57].

## 5. Conclusions

In this study, nitrogen (N) deposition significantly promoted the growth and root elongation of both Chinese fir and moso bamboo. Chinese fir exhibited higher tolerance to N deposition and stronger root morphological plasticity in response to N than moso bamboo. Chinese fir adapted to N deposition by developing root systems with higher fine root density, greater branching intensity and angle, and a morphology approaching dichotomous branching. In the first half of the growing season, moso bamboo allocates more biomass and N to aboveground parts, whereas in the second half, it allocates more biomass and P to roots to adapt to N deposition. Although N deposition enhanced biomass and N accumulation in both species in the short term, it caused substantial consumption of phosphorus (P) and soluble sugars (SS), leading to N/P imbalance and proving unfavorable for their long-term growth and development. Under conditions of P and SS deficiency, Chinese fir tended to preferentially allocate SS to leaves, while moso bamboo prioritized the allocation of P and SS to roots to meet the growth needs of their respective key functional organs. The research identifies highly adaptive root morphological and architectural patterns, demonstrating that optimizing the allocation of elements and photosynthates and avoiding elemental balance risks represent critical survival mechanisms for subtropical tree species under intensified N deposition. This study reveals the distinct adaptive strategies of two coexisting tree species under N deposition, providing a scientific basis for maintaining the ecological stability of mixed forests.

## Figures and Tables

**Table 1:** Effects of treatments, periods, species, and their interactions on indicators.

Indicators	Treatments	Periods	Species	Treatments × Periods	Treatments × Species	Periods × Species	Treatments × Periods × Species
RB	4.02 *	736.65 **	1457.26 **	2.776 *	2.139	345.61 **	1.66
SB	3.93 *	369.19 **	1428.21 **	4.06 *	2.79 *	244.06 **	3.33 *
LB	24.59 **	832.05 **	3502.02 **	14.38 **	23.26 **	733.01 **	13.73 **
Root–shoot ratio	15.54 **	724.44 **	7128.97 **	19.67 **	4.56 **	335.20 **	39.49 **
RL	19.19 **	548.63 **	328.85 **	15.30 **	1.69	7.81 **	1.12
RSA	6.60 **	487.56 **	1020.43 **	4.67 **	5.51 **	118.14 **	4.61 **
RD	1.64	94.57 **	1691.84 **	2.58	3.59 *	34.75 **	1.52
SRL	9.06 **	223.10 **	701.00 **	2.91 *	13.135 **	115.35 **	0.94
RT	8.52 **	830.04 **	18.71 **	3.924 *	19.91 **	37.41 **	16.03 **
FD	1.82	187.85 **	835.32 **	1.05	1.03	39.05 **	0.594
RBA	1.32	0.29	243.94 **	0.22	0.31	0.45	0.27
TI	5.15 **	3.32	21.42 **	0.51	0.31	0.32	0.61
Leaves N	112.24 **	11.94 **	354.04 **	50.01 **	17.35 **	12.65 **	0.92
Stems N	70.96 **	5.90 *	76.71 **	6.67 **	12.95 **	4.34 *	1.34
Roots N	57.35 **	0.04	95.93 **	9.57 **	2.04	4.86 *	1.61
Leaves P	60.09 **	13.50 **	228.90 **	1.61	0.93	44.77 **	2.19
Stems P	59.99 **	36.08 **	22.99 **	1.04	1.55	1.42	0.99
Roots P	11.82 **	28.89 **	5.60 *	0.01	3.36 *	2.61	0.14
Leaves N/P	155.47 **	3.98	0.61	21.00 **	22.91 **	50.18 **	2.30
Stems N/P	118.01 **	35.54 **	101.00 **	7.33 **	18.28 **	2.55	1.63
Roots N/P	70.81 **	22.37 **	22.35 **	5.45 **	5.97 **	7.06 **	1.12
Leaves SS	11.15 **	23.52 **	1514.1 **	1.00	0.51	20.19 **	0.14
Stems SS	40.40 **	2.98	164.67 **	5.98 **	6.06 **	15.24 **	3.83
Roots SS	25.66 **	0.55	539.51 **	9.07 **	24.60 **	0.70	5.63 **
Leaves ST	24.81 **	27.07 **	272.65 **	0.684	0.647	4.91 *	0.10
Stems ST	13.84 **	56.33 **	1853.57 **	1.04	5.05 **	0.003	0.397
Roots ST	47.24 **	19.38 **	366.57 **	1.04	7.84 **	2.13	0.76
Leaves SS/ST	50.18 **	62.01 **	1778.31 **	3.73 *	5.38 **	19.62 **	1.14
Stems SS/ST	73.57 **	54.27 **	1862.53 **	6.42 **	16.50 **	1.50	5.42 **
Roots SS/ST	112.23 **	33.80 **	1253.38 **	13.52 **	33.02 **	8.66 **	4.83 **
Leaves N accumulation ratio	5.03 **	218.89 **	2322.66 **	0.71	3.47 *	34.47 **	5.49 **
Stems N accumulation ratio	0.77	5.67 **	1.23	4.06 *	3.01 *	0.10	0.59
Roots N accumulation ratio	8.03 **	370.99 **	3069.56 **	3.03 *	0.04	48.25 **	8.04 **
Leaves P accumulation ratio	9.12 **	314.66 **	2563.20 **	6.57 **	8.12 **	606.00 **	6.34 **
Stems P accumulation ratio	6.77 **	424.20 **	28.00 **	6.41 **	5.09 **	506.11 **	4.68 **
Roots P accumulation ratio	8.48 **	657.11 **	127.79 **	8.22 **	9.725 **	473.75 **	11.02 **

Note: the values are F-values; *: *p* < 0.05; **: *p* < 0.01; RB: root biomass, SB: stem biomass, LB: leaf biomass, RL: root length, RSA: root surface area, RD: root average diameter, SRL: specific root length, RT: root tips, FD: fractal dimension; RBA: root branching angle, TI: root topological index, N: nitrogen, P: phosphorus, N/P: nitrogen/phosphorus ratio, SS: soluble sugar, ST: starch, SS/ST: soluble sugar/starch ratio.

**Table 2:** Effect of N deposition on the nutrient element allocation of moso bamboo and Chinese fir seedlings.

Periods	Nutrient Content and Ratio	Treatments	Moso Bamboo			Chinese Fir		
			Leaves	Stems	Roots	Leaves	Stems	Roots
July	P (mg·g^−1^)	N0	1.174 ± 0.075 a	0.626 ± 0.034 a	0.696 ± 0.037 a	1.090 ± 0.057 a	0.605 ± 0.022 a	0.739 ± 0.037 a
		N1	1.098 ± 0.101 ab	0.588 ± 0.048 ab	0.639 ± 0.061 a	0.983 ± 0.086 ab	0.528 ± 0.035 b	0.667 ± 0.022 b
		N2	0.966 ± 0.055 bc	0.537 ± 0.032 b	0.624 ± 0.025 a	0.867 ± 0.028 b	0.500 ± 0.020 b	0.641 ± 0.041 bc
		N3	0.950 ± 0.047 c	0.516 ± 0.040 b	0.632 ± 0.069 a	0.750 ± 0.023 c	0.451 ± 0.029 c	0.587 ± 0.056 c
	N (mg·g^−1^)	N0	21.96 ±1.23 a	7.37 ± 0.55 a	9.85 ± 0.27 a	14.64 ±0.55 c	7.56 ± 0.55 b	10.86 ± 0.41 c
		N1	22.26 ± 1.28 a	7.45 ± 0.39 a	10.18 ± 0.49 a	16.20 ± 0.79 b	8.89 ± 0.80 ab	12.02 ± 0.57 b
		N2	22.84 ± 1.03 a	8.08 ± 0.75 a	10.75 ± 0.66 a	17.14 ± 0.42 b	10.51 ± 1.29 a	12.42 ± 0.77 ab
		N3	21.86 ± 0.91 a	8.01 ± 0.39 a	10.52 ± 0.82 a	18.78 ± 0.53 a	11.06 ± 0.68 a	13.42 ± 0.82 a
	N/P	N0	18.73 ±0.96 b	11.77 ± 0.72 b	14.18 ± 0.65 b	13.46 ±0.93 d	12.49 ± 0.61c	14.74 ± 1.17 c
		N1	20.39 ± 2.02 ab	12.69 ± 1.01 b	16.01 ± 1.17 a	16.55 ± 1.30 c	16.90 ± 1.66 b	18.01 ± 0.72 b
		N2	23.72 ±1.87 a	15.02 ± 0.69 a	17.22 ± 0.64 a	19.79 ± 0.59 b	21.04 ± 2.40 a	19.41 ± 1.18 b
		N3	23.06 ± 1.57 a	15.55 ± 0.80 a	16.73 ± 1.62 a	25.06 ± 0.93 a	24.66 ± 2.65 a	23.01 ± 2.274 a
December	P (mg·g^−1^)	N0	1.270 ± 0.090 a	0.602 ± 0.057 a	0.613 ± 0.066 a	0.872 ± 0.056 a	0.574 ± 0.031 a	0.700 ± 0.040 a
		N1	1.075 ± 0.047 b	0.491 ± 0.025 b	0.571 ± 0.045 a	0.797 ± 0.034 ab	0.493 ± 0.031 b	0.619 ± 0.028 b
		N2	1.046 ± 0.054 b	0.480 ± 0.046 b	0.555 ± 0.071 a	0.740 ± 0.075 bc	0.460 ± 0.022 b	0.595 ± 0.050 bc
		N3	0.976 ± 0.072 b	0.473 ± 0.029 b	0.550 ± 0.031 a	0.668 ± 0.069 c	0.407 ± 0.015 c	0.547 ± 0.042 c
	N (mg·g^−1^)	N0	16.35 ± 0.72 c	6.80 ± 0.41 c	8.84 ± 0.48 c	10.48 ± 1.02 d	7.06 ± 0.43 c	9.67 ± 0.70 c
		N1	20.10 ± 0.98 b	8.30 ± 0.51 b	10.05 ± 0.37 b	14.60 ± 0.54 c	8.12 ± 0.67 b	11.30 ± 0.43 b
		N2	21.35 ± 1.75 ab	8.83 ± 0.81 ab	11.33 ± 0.75 ab	18.30 ± 1.45 b	11.03 ± 1.10 a	12.84 ± 0.59 a
		N3	24.18 ± 1.55 a	9.95 ± 0.42 a	12.33 ± 1.32 a	23.48 ± 1.50 a	12.04 ± 0.79 a	13.56 ± 0.95 a
	N/P	N0	12.91 ± 1.00 c	11.40 ± 1.56 c	14.57 ± 2.05 c	12.01 ± 0.64 d	12.31 ± 0.94 d	13.83 ± 1.18 d
		N1	18.72 ± 1.04 b	16.97 ± 1.88 b	17.67 ± 1.72 bc	18.36 ± 1.30 c	16.54 ± 1.76 c	18.31 ± 1.34 c
		N2	20.46 ± 1.95 ab	18.57 ± 2.93 ab	20.57 ± 1.73 ab	24.99 ± 3.77 b	24.07 ± 3.19 b	21.70 ± 1.88 b
		N3	24.90 ± 2.68 a	21.11 ±1.89 a	22.76 ± 2.52 a	35.41 ± 3.63 a	29.66 ± 2.42 a	24.94 ± 3.09 a

Note: a, b, c, d—different letters indicate significant differences among different treatments (*p* < 0.05). The values are the means and standard errors. N: nitrogen, P: phosphorus, N/P: nitrogen/phosphorus ratio.

**Table 3:** Effect of N deposition on the non-structural carbohydrate allocation of moso bamboo and Chinese fir seedlings.

Periods	NSCs Content and Ratio	Treatments	Moso Bamboo			Chinese Fir		
			Leaves	Stems	Roots	Leaves	Stems	Roots
July	soluble sugar (%)	N0	8.69 ± 0.50 a	8.20 ± 0.56 a	6.67 ± 0.37 a	14.32 ± 0.85 a	10.78 ± 0.50 a	12.08 ± 0.93 a
		N1	7.41 ± 0.75 b	7.36 ± 0.44 b	7.09 ± 0.39 a	13.46 ± 0.85 a	10.00 ± 0.48 ab	10.85 ± 1.24 ab
		N2	7.72 ± 0.28 b	7.17 ± 0.23 b	7.20 ± 0.50 a	13.49 ± 0.91 a	9.59 ± 1.01 b	10.77 ± 0.38 b
		N3	7.20 ± 0.55 b	6.95 ± 0.54 b	6.80 ± 0.40 a	13.58 ± 1.01 a	9.23 ± 0.75 b	10.23 ± 0.73 b
	Starch (%)	N0	8.08 ± 0.53 c	11.81 ± 0.99 b	8.46 ± 0.51 c	5.92 ± 0.46 b	6.86 ± 0.40 a	6.55 ± 0.42 c
		N1	8.72 ± 0.79 b	12.08 ± 0.61 ab	9.93 ± 0.62 b	6.65 ± 0.52 ab	6.88 ± 0.34 a	7.28 ± 0.48 bc
		N2	8.92 ± 0.38 ab	13.50 ± 0.37 a	10.53 ± 1.20 ab	6.93 ± 0.45 a	7.04 ± 0.26 a	7.63 ± 0.37 ab
		N3	9.43 ± 0.65 a	13.55 ± 0.81 a	11.73 ± 1.05 a	6.86 ± 0.58 a	7.68 ± 0.70 a	8.33 ± 0.41 a
	Sugar/starch	N0	1.077 ± 0.044 a	0.695 ± 0.035 a	0.791 ± 0.068 a	2.427 ± 0.194 a	1.574 ± 0.082 a	1.845 ± 0.112 a
		N1	0.851 ± 0.068 b	0.611 ± 0.046 b	0.715 ± 0.027 b	2.025 ± 0.076 b	1.454 ± 0.087 ab	1.491 ± 0.129 b
		N2	0.867 ± 0.053 b	0.532 ± 0.026 c	0.687 ± 0.040 b	1.950 ± 0.121 b	1.360 ± 0.094 b	1.387 ± 0.062 bc
		N3	0.764 ± 0.042 c	0.515 ± 0.050 c	0.582 ± 0.045 c	1.990 ± 0.225 b	1.207 ± 0.123 b	1.230 ± 0.098 c
December	soluble sugar (%)	N0	8.76 ± 0.55 a	9.08 ± 0.31 a	7.28 ± 0.38 a	16.12 ± 0.81 a	11.81 ± 1.12 a	13.98 ± 1.59 a
		N1	7.82 ± 0.58 b	8.33 ± 0.38 b	7.32 ± 0.32 a	15.60 ± 0.76 a	10.47 ± 0.74 a	11.89 ± 0.828 b
		N2	7.63 ± 0.38 b	8.20 ± 0.53 b	7.39 ± 0.46 a	14.95 ± 1.08 a	8.49 ± 0.77 b	9.50 ± 0.36 c
		N3	7.07 ± 0.60 b	7.40 ± 0.49 c	6.83 ± 0.45 a	14.64 ± 0.97 a	7.53 ± 0.56 b	8.31 ± 0.51 d
	Starch (%)	N0	7.02 ± 0.53 b	11.07 ± 0.69 c	7.45 ± 0.65 c	5.34 ±0.35 c	6.05 ± 0.49 a	5.80 ± 0.46 b
		N1	7.82 ± 0.34 a	11.41 ± 0.62 b	9.60 ± 0.80 b	6.28 ± 0.42 b	6.09 ± 0.27 a	6.59 ± 0.25 a
		N2	8.11 ± 0.60 a	12.13 ± 0.38 b	10.71 ± 1.03 ab	6.47 ± 0.23 b	6.16 ± 0.56 a	6.81 ± 0.36 a
		N3	8.72 ± 0.53 a	12.39 ± 0.61 a	11.08 ± 0.81 a	6.87 ± 0.51 a	6.27 ± 0.40 a	7.00 ± 0.49 a
	Sugar/starch	N0	1.249 ± 0.028 a	0.822 ± 0.037 a	0.981 ± 0.064 a	3.027 ± 0.023 a	1.954 ± 0.135 a	2.415 ± 0.269 a
		N1	0.999 ± 0.040 b	0.732 ± 0.052 b	0.766 ± 0.069 b	2.491 ± 0.192 b	1.720 ± 0.082 b	1.807 ± 0.149 b
		N2	0.942 ± 0.034 b	0.676 ± 0.028 b	0.692 ± 0.030 bc	2.316 ± 0.233 b	1.381 ± 0.112 c	1.397 ± 0.065 c
		N3	0.811 ± 0.059 c	0.597 ± 0.027 c	0.618 ± 0.056 c	2.137 ± 0.136	1.206 ± 0.138 c	1.190 ± 0.095 d

Note: a, b, c, d—different letters indicate significant differences among different treatments (*p* < 0.05). The values are the means and standard errors.

## Data Availability

The original contributions presented in this study are included in the article. Further inquiries can be directed to the corresponding author.
